# Sensitivity of the Dengue Surveillance System in Brazil for Detecting Hospitalized Cases

**DOI:** 10.1371/journal.pntd.0004705

**Published:** 2016-05-18

**Authors:** Giovanini Evelim Coelho, Priscila Leite Leal, Matheus de Paula Cerroni, Ana Cristina Rocha Simplicio, João Bosco Siqueira

**Affiliations:** 1 National Dengue Control Program, Ministry of Health, Brasilia, Brazil; 2 Institute of Tropical Pathology and Public Health, Federal University of Goiás, Goiania, Brazil; Duke-NUS GMS, SINGAPORE

## Abstract

We evaluated the sensitivity of the dengue surveillance system in detecting hospitalized cases in ten capital cities in Brazil from 2008 to 2013 using a probabilistic record linkage of two independent information systems hospitalization (SIH-SUS) adopted as the gold standard and surveillance (SINAN). Sensitivity was defined as the proportion of cases reported to the surveillance system amid the suspected hospitalized cases registered in SIH-SUS. Of the 48,174 hospitalizations registered in SIH-SUS, 24,469 (50.7%) were reported and registered in SINAN, indicating an overall sensitivity of 50.8% (95%CI 50.3–51.2). The observed sensitivity for each of the municipalities included in the study ranged from 22.0% to 99.1%. The combination of the two data sources identified 71,161 hospitalizations, an increase of 97.0% over SINAN itself. Our results allowed establishing the proportion of underreported dengue hospitalizations in the public health system in Brazil, highlighting the use of probabilistic record linkage as a valuable tool for evaluating surveillance systems.

## Author Summary

This manuscript address essential issues regarding the current and future challenges of a dengue surveillance system. Dengue fever is one of the major public health threats in a large area of the world, including Brazil which represents around 70% of the cases in the Americas. The dengue surveillance system in Brazil was established in 1986, but evaluations of this surveillance system were rarely conducted. The need for accurate data is of paramount importance due to not only the increase of severe cases in the past decade, but also to monitor the impact of a vaccine as soon as it occurs. The evaluation of the sensitivity of surveillance system is a challenge, due to the lack of a gold standard. In our study, we took advantage of two very well structured and independent information systems with nationwide coverage. The hospitalization information system was defined as the gold standard for hospitalized cases in the public health system and compared to the notifiable diseases information system using a probabilistic record linkage. Therefore, we were able to evaluate the sensitivity of the dengue surveillance system in detecting hospitalized cases in ten capital cities from 2008 to 2013 in Brazil.

## Introduction

Dengue is the most important arboviral disease in the world due to the associated morbidity, mortality and economic burden [1–4]. In Brazil, the disease has become a major public health challenge, with 5.8 million probable cases, 555 thousand hospitalizations and 3,000 deaths reported from 2002 to 2014. During this period, noteworthy epidemiologic shifts were observed in the country, including an increase in the number of smaller cities experiencing transmission, changes in affected age groups and increases in the proportion of severe cases [5–8].

Dengue fever is a mandatorily reportable disease in Brazil since the reintroduction of the virus to the country in 1986 [7]. The dengue surveillance system relies on passive reporting from healthcare facilities (outpatient and hospital), with uniform standardized forms used throughout the country. The data from these forms are entered into the National Reportable Disease Information System—SINAN (Sistema de Informação de Agravos de Notificação), which is the main source for dengue related information in Brazil. As expected, passive surveillance likely results in under-reporting, especially with regard to undifferentiated febrile and atypical forms of dengue [9,10]. This limitation may lead to an underestimated burden of the disease, which can result in inappropriate allocation of resources for prevention and control activities[11–13].

The continuous evaluation of surveillance systems with respect to their attributes, is critical for maximizing the efficacy and the utility of such systems and for producing more reliable indicators [14]. The sensitivity of a surveillance system is the capacity to identify cases of the disease and, therefore, is a crucial attribute for a system to reach its goals. However, the evaluation of the sensitivity is usually a challenge due the lack of a gold standard to provide the true number of cases for comparison [15]. The record linkage of different information systems is an alternative to improve disease estimates and evaluate the sensitivity of surveillance systems [16–18]. Hospitalizations in the public health system in Brazil (National Unified Health System-SUS) are registered in a specific information system that is independent of the surveillance system. The objective of this study was to estimate the sensitivity of the national dengue surveillance system (SINAN) for detecting hospitalized dengue cases in the National Unified Health System (SUS) in 10 state capitals between 2008 and 2013 in Brazil.

## Methods

### Ethics Statement

This study was approved by the Committee for Ethics in Research of the Federal University of Goiás in accordance with the ethics principles established in Resolution 466/12 of the National Council for Health of Brazil and all data analyzed were anonymized.

This an observational, descriptive and cross-sectional epidemiologic study based on the probabilistic record linkage between the databases of the National Unified Health System’s Hospital Information System (we use the Portuguese acronym SIH-SUS) and the National Reportable Disease Information System (SINAN).

Study area and period: We selected ten state capitals located in the four dengue endemic regions of Brazil for the study. These municipalities contributed 10% of the total hospitalized dengue cases from 2008 to 2013, the study period. Although the municipalities vary in size, they were similar with regard to epidemiological aspects of dengue such as the historical circulation of the four viral serotypes (DENV 1, 2, 3, 4) and the occurrence of epidemics. The selected capitals were the following (city (state)): North Region—Manaus (AM), Boa Vista (RR); Northeast Region–Fortaleza (CE), Natal (RN), São Luis (MA), Teresina (PI); Southeast Region—Rio de Janeiro (RJ), Belo Horizonte (MG); and Central-West Region–Goiânia (GO), Campo Grande (MS).

### Data Sources

Dengue hospitalized cases: The organization of the Public Health System (SUS) in Brazil has been described in detail elsewhere [19]. Briefly, this system provides universal healthcare to all persons residing in Brazil, outpatient and inpatient, at no charge to patients. Approximately 70% of inpatient medical services in the country are provided by SUS [20]. Hospitalizations in SUS requires completion of a standard form that captures patients’ personal data, symptoms, and the initial diagnosis coded according to the 10th revision of the International Classification of Disease (ICD-10). This form and further information on diagnoses, treatment, test results, and billing are the main data recorded by the SIH-SUS, which is an administrative database standardized throughout Brazil. This system captures data on all hospitalizations paid by the public health system for public and contracted hospitals. The resulting data is checked and validated by local health authorities and subsequently transmitted to regional and national levels. For this study, we extracted the records for suspected dengue cases that were hospitalized in SUS, using admission ICD10 codes A90 and A91 for Dengue and Dengue Hemorrhagic Fever. We used the SIH-SUS database, updated in January 2014, for the years 2008 to 2013.

Reported dengue Cases: The organization of the surveillance system has been previously described [7]. In summary, dengue is a mandatorily reportable disease and the system relies on the notification of all suspected cases at public and private health facilities based on the attending clinician’s initial clinical diagnosis (not laboratory confirmed). SINAN is the official information system for entering and processing the data for reported dengue cases throughout Brazil. It uses uniform standardized forms that capture data related to patient identification as well as the main characteristics of the illness. Data on patient hospitalization during each dengue disease episode is also recorded and this procedure is independent of SIH-SUS routines. During the study period, the Ministry of Health (MoH) of Brazil adopted the case definitions proposed by the Pan American Health Organization (PAHO/WHO) for suspected and confirmed cases of Dengue Fever (DF) and Dengue Hemorrhagic Fever (DHF). Additionally, the MOH adopted an intermediate final classification “dengue with complications” (DwC) that includes all the cases that did not fulfill the DHF diagnosis criteria and ones for which the DF classification was not satisfactory due to the severity of the clinical and laboratory outcomes presented. The final classification of each case is only performed after the patient’s discharge or conclusion of the case investigation.

Identification and deletion of duplicated records are conducted at the local level using SINAN’s automated routine and the resulting data is transferred to state and federal levels. Failure to transmit data from the local level for a period of two months is penalized by cancellation of financial resources destined to the municipality.

### Data Analysis

Initially we identified and excluded duplicate records and inconsistencies in both databases. During the process of standardization of databases a total 25,047 duplicate records were excluded (23,232 from SINAN and 1,815 from SIH-SUS). Using the cleaned databases, we generated descriptive findings of dengue related hospitalizations by gender, age group and diagnosis according to ICD-10 in each of the two systems. The number of dengue-related hospitalizations in each state capital derived from SIH-SUS was defined as the gold standard for the subsequent sensitivity analysis.

A probabilistic record linkage of all dengue related-hospitalized recorded in SIH-SUS and all reported cases in SINAN was performed. We used RecLinkIII software, which implements the probabilistic record linkage methodology and is widely used for this purpose in Brazil [21,22]. The output of this software includes a score for the links formed to assess the agreement and disagreement of the variables selected for the linkage. The higher the score, the greater the probability of finding a true matched pair.

Prior to record linkage, both databases underwent a pre-processing stage of quality analysis to minimize errors and increase the likelihood of finding matched records. These procedures comprised mainly standardization of the variables selected as matching and/or blocking variables.

The record linkage process consisted of the following steps:

1. The following matching variables were included in the probabilistic record linkage: patients’ name, date of birth, mother’s name, and city of residence.
2. Gender was used as a blockage variable. This step created mutually exclusive blocks in the two databases for a greater speed in the linkage procedure.
3. The resulting score of the linkage was used to attribute the status of the pair as follows: “true” (scores from 19.3 to 38.1), “questionable” (scores from 13.1 to 19.3), and “non-pair” (scores<13.1).
4. “Questionable” pairs were manually reviewed with the objective of reclassification to “true” or “non-pair”. A similar procedure was also applied to a randomly selected sample of “true” pairs and “non-pairs” for quality control.

The sensitivity of the surveillance system for detecting hospitalized cases in SIH-SUS was calculated using two approaches. In method 1, the numerator consisted of the total number of true pairs, which were also described as being hospitalized in SINAN; the denominator consisted of the total number of dengue related hospitalizations in SIH-SUS. In method 2, the numerator consisted of the total number of true pairs regardless of the reported hospitalization status in SINAN; the denominator consisted, again, of the total number of dengue related hospitalizations in SIH-SUS. To estimate the overall number of hospitalization was adopted the formula: (SINAN hospitalization X SIH-SUS)/Matched pairs[23]

Data was processed and analyzed using Tabwin, Reclink III version 3.1.6 and Microsoft Office 2010.

## Results

**Fig 1:**
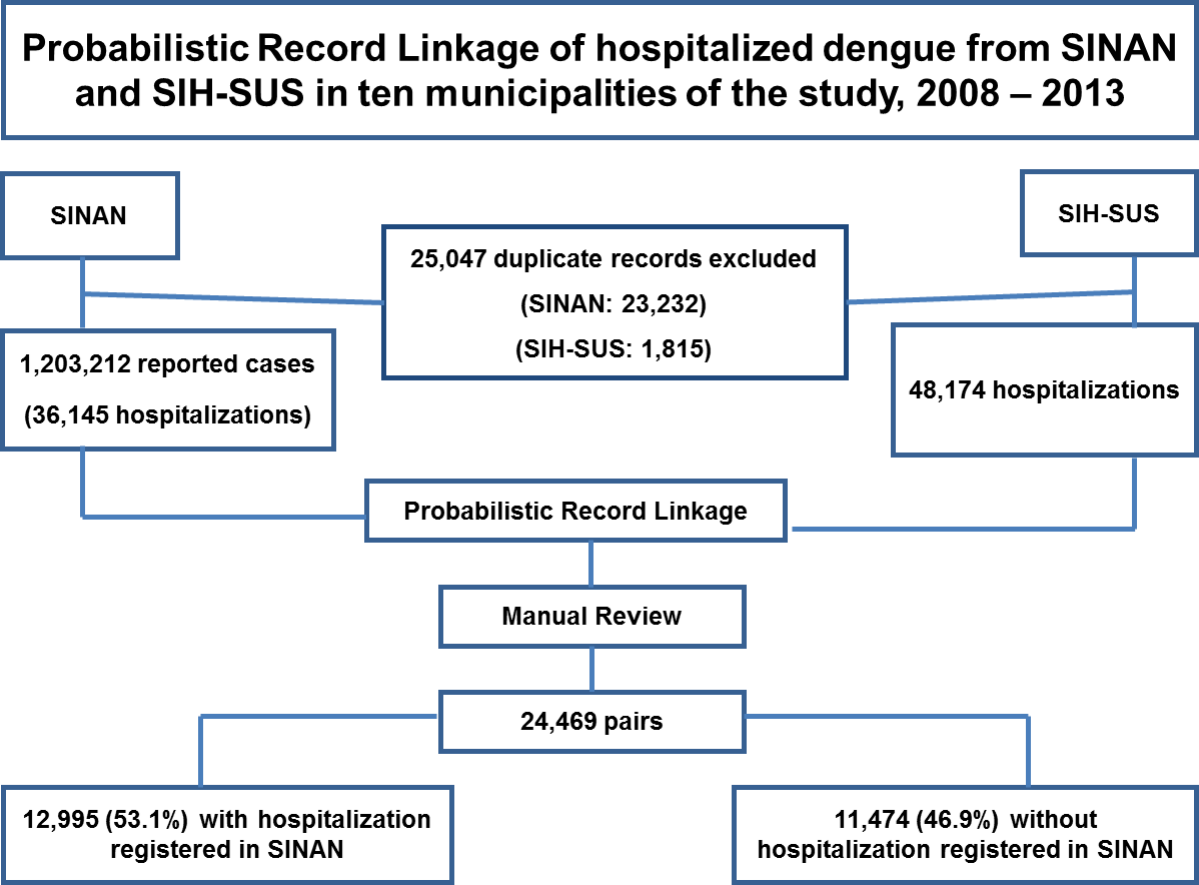
Probabilistic record linkage of hospitalized dengue cases in the Hospitalization Information System (SIH-SUS) and the reported dengue cases from the Notifiable Diseases Information System (SINAN) and in ten municipalities of the study, Brazil, 2008–2013.

During the study period, 1,203,212 suspected dengue cases were reported in the 10 selected municipalities and of these 36,145 (3.0%) were hospitalized according to SINAN. In SIH-SUS, 48,174 dengue hospitalizations were registered during the same period. Overall, the number of hospitalizations recorded in SIH-SUS was 33.3% higher than those recorded in SINAN (Fig 1).

However, this pattern was not observed in the municipalities of São Luis (MA), Rio de Janeiro (RJ) and Campo Grande (MS) where the number of hospitalizations was higher in SINAN exceeded those recorded in SIH-SUS by 5,207 (31.2%) (S1 Table).

The distribution of hospitalizations by sex showed a similar pattern in both information systems, with females accounting for about 51% of the records. The proportion of hospitalized cases in children under 15 years was higher in SIH-SUS (45.0%) compared to SINAN (38.5%). This pattern was observed in most capitals except in Boa Vista (RR) and Teresina (PI) where the proportion of hospitalizations in children were 55.8% and 29.6% in SINAN and 44.0% and 28.3% in SIH-SUS respectively.

Patients with DF accounted for 43.4% and 83.1% of hospitalizations in SINAN and SIH-SUS, respectively. Almost twice as many hospitalizations due to suspected DHF were observed in SIH-SUS compared with those in SINAN: 8,123 (17.0%) vs. 3,346 (9.2%) records, respectively. However, 14,030 hospitalizations (38.8%) were classified as DwC in SINAN, highlighting that some of these cases may include suspected DHF inpatients. Only in Boa Vista (RR) there was a higher number of hospitalizations due to DHF in SINAN (Table 1).

**Table 1:** Hospitalizations recorded in the SINAN and SIH-SUS information systems by sex age and classification, in ten municipalities in Brazil, 2008–2013.

General characteristics	2008–2013			
	SINAN		SIH-SUS	
	N	(%)	N	(%)
**Hospitalizations**				
Yes	36,145	(100.0)	48,174	(100.0)
**Gender**				
Female	18,570	(51.8)	24,54	(51.4)
**Age**				
<15 years	13,918	(38.5)	21,702	(45.0)
15 a 59 years	17,130	(47.4)	22,487	(46.7)
60 and more	3,261	(9.0)	3,984	(8.3)
Ignored	1,836	(5.1)	1	(0.002)
Final Classification*				
DF	15,681	(43.4)	40,051	(83.1)
DwC	14,030	(38.8)	-	-
DHF/DSS	3,346	(9.2)	8,123	(16.9)
Probable cases	705	(2.0)		
Discarded	2,383	(6.6)	-	-

*DF- Dengue Fever; DwC–Dengue with complications; DHF–Dengue Hemorrhagic Fever; DSS–Dengue shock syndrome

The probabilistic record linkage identified 24,469 records common to both databases. Overall, among the total number of pairs found, 12,995 (53.1%) had the field for the variable “hospitalization” completed in the SINAN record, with the highest proportion observed in 2008 (26.6%) (Table 2).

**Table 2:** Number of cases hospitalized in SINAN, SIH-SUS and the sensitivity of the dengue surveillance system in detecting hospitalized cases I n ten municipalities in Brazil, 2008–2013.

Information Source	2008	2009	2010	2011	2012	2013	Total
	N	N	N	N	N	N	N
SINAN Hospitalization	11,108	2,177	6,514	9,094	3,758	3,494	36,145
SIH-SUS	18,251	3,455	8,202	10,050	4,836	3,380	48,174
Pairs SINAN and SIH-SUS	7,781	1,425	5,116	4,299	3,174	2,674	24,469
With hospitalization registered in SINAN	3,462	287	3,055	2,942	1,832	1,417	12,995
Without hospitalization registered in SINAN	4,319	1,138	2,061	1,357	1,342	1,257	11,474
Sensitivity method 1* **% (IC95%)**	19(18.4–19.4)	8.3 (7.4–9.3)	37.2(36.2–38.3)	29.3(28.4–30.2)	37.9(36.5–3.3)	41.9(40.2–43.6)	27.0(26.6–27.4)
Sensitivity Method 2^†^ **% (IC95%)**	42.6(41.9–43.3)	41.2 (39.6–42.9)	62.4(6.3–63.4)	42.8(41.8–43.7)	65.6(64.3–66.9)	79.1(77.7–80.5)	50.8 (50.3–51.2)
Total Estimated Hospitalizations^¥^	26,054	5,278	10,443	21,258	5,726	4,416	71,161

*Numerator: total number of true pairs that were also reported as being hospitalized in SINAN; Denominator: total number of dengue related hospitalizations in SIH-SUS

^†^ Numerator: total number of true pairs regardless of the reported hospitalization status in SINAN; Denominator: total number of dengue related hospitalizations in SIH-SUS.

^¥^Total Estimated Hospitalizations: calculated using the formula: (SINAN hospitalization X SIH-SUS)/Matched Pairs[23]

However, different results were observed for the years of 2010 and 2009, with, respectively, 23.5% and 2.2% of the pairs with information on hospitalization available in SINAN.

The combination of the two systems allowed identification of 71,161 hospitalizations, which represented increases of 97.0% and 47.7% in the number previously registered in SINAN (36,145) and SIH- SUS (48,174), respectively (Table 2).

The sensitivity of the surveillance system in detecting cases hospitalized in SUS was 27.0% (95% CI 26.6 to 27.4), when considering only records with information regarding hospitalization in both systems (method 1). Using this approach, the lowest sensitivity was observed in 2009 at 8.3% (95%CI: 7.4 to 9.3) and the highest in 2013 at 41.9% (95% CI 40.2–43.6). Among the municipalities the highest and lowest sensitivities were observed in Campo Grande (MS) at 78.5% (95%CI: 67.5 to 86.6) in 2012 and 0% in 2008, when none of the 15 hospitalizations were registered in SINAN.

**Fig 2:**
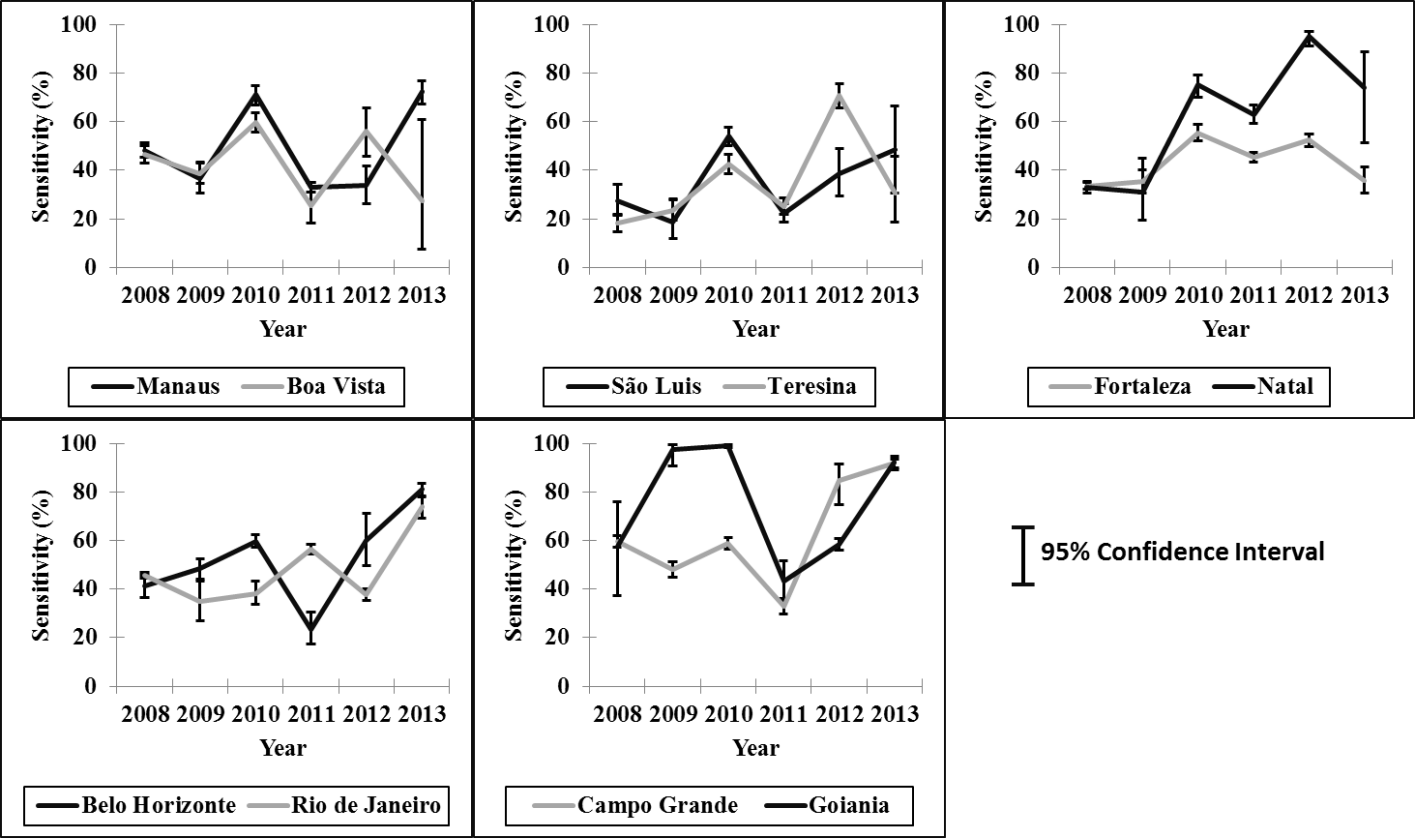
Sensitivity of the dengue surveillance system in detecting hospitalized dengue cases in ten municipalities in Brazil, 2008–2013.

The sensitivity of the surveillance system including all records in SINAN regardless of the hospitalization status (method 2) was almost twice as high as that calculated by the first approach. Using this method, the cumulative sensitivity was 50.8% (95%CI: 50.3–51.2), with the lowest value observed in 2009 at 41.2% (95%CI: 39.6–42.9) and the highest in 2013 at 79.1% (95%CI: 77.7–80.4). Among the municipalities, the highest value was observed in Campo Grande (MS) at 99.2% (95%CI: 98.3–99.6) in 2010 and the lowest in Teresina (PI) at 18.1% (95%CI: 14.8–21.9) in 2008 (Table 2; Fig 2).

The comparison of matched pairs according to the initial clinical suspicion of dengue from SIH-SUS and the final classification according to the surveillance system is presented in Table 3.

**Table 3:** Pairs identified by the probabilistic record linkage according to the initial clinical assessment in SIH-SUS and final case classification in SINAN in the 10 municipalities of the study, Brazil, 2008–2013.

SINAN Final Classification*	SIH-SUS Initial Clinical Assessment*	
	DF (A90)	DHF (A91)
	N (%)	N (%)
DF	11,482 (57.5)	1,604 (35.5)
DwC	3,456 (17.2)	1,407 (31.2)
DHF/DSS	975 (4,9)	685 (15.1)
Not Classified (missing information)	2,221 (11.2)	626 (13.9)
Discarded	1,820 (9.2)	193 (4.3)
Total	19,954 (100)	4,515 (100)

*DF- Dengue Fever; DwC–Dengue with complications; DHF–Dengue Hemorrhagic Fever; DSS–Dengue shock syndrome

Among the 4,515 pairs hospitalized in SIH-SUS and classified as DHF (A91), 35.5% (1604) were classified in SINAN as DF, 31.2% (1,407) as DwC and 15.1% as DHF / DSS. Of the 19,954 patients hospitalized with a classification of DF (A90), 57.5% (11,482) had a final classification of DF, 17.2% (3,456) were reclassified as DwC and 4.9% DHF and DSS. The percentage of pairs that lacked a classification by the surveillance system was 11.6%.

## Discussion

In this study, we demonstrated the occurrence of a larger number of hospitalized dengue fever cases in Brazil than that captured in the national surveillance system (SINAN). The use of probabilistic record linkage of SINAN data and the national hospitalization system (SIH-SUS) database expanded the estimate of dengue hospitalizations by over 49.2% (35,016) hospitalizations in the ten cities of the study, compared to the data available from SINAN alone.

The dengue surveillance system should capture all reported cases from both public and private health systems. We therefore expected that the total number of cases in the SINAN surveillance database would exceed that of SIH-SUS, which only covers the public health system. However, this was not observed in general, except for three municipalities, where the higher number of hospitalizations observed in SINAN when compared to SIH-SUS could reflect an improved participation from private hospitals in surveillance activities. The surveillance system did not allow identifying if the reporting health unit is public or private, but the inclusion of this data would improve the quality and representativeness of the surveillance system.

The adoption of two different approaches to evaluate the sensitivity allowed a more comprehensive analysis of the current operational aspects of the surveillance system. Most hospitalized suspected cases of dengue were not reported to the surveillance system or were reported before hospitalization. Only 12,995 (53%) of the reported dengue cases had data on the hospitalization status in the surveillance information system. The completion of the appropriate data field for hospitalization status in the SINAN surveillance form would enable the surveillance system to capture the burden of the disease and its trends over time.

Interpretation of the results of our analysis for 2008 and 2009 requires caution. A new version of SINAN software was implemented nationwide in January 2007. The updated version did not allow filling the field for the hospitalization variable for cases classified as DF, even if the data was available from the investigation form. As this limitation was a technical oversight, it was corrected with a software patch in late 2009. Since the attributes of a surveillance system are intrinsically interconnected, the sensitivity in this case was influenced by the limited capacity to adapt to changing information needs or operating conditions, in other words, the flexibility of the system.

Although SINAN and SIH-SUS are completely independent of each other, the distribution of sex and age groups of hospitalized patients was very similar for the cases captured by each of the two systems. However, the observed differences in initial clinical diagnosis at the moment of the admission and the final classification performed after the full course of the illness highlight some of the difficulties discussed over time in the disease classification [24,25]. This disagreement reinforces the importance of the surveillance routines adopted by the Brazilian surveillance system. The final classification of cases reflects the investigation conducted locally by public health professionals based on chart review and clinical and laboratory findings, in accordance national guidelines of the Ministry of Health[26]. Annually, 30 to 40% of reported cases are discarded by the surveillance system in Brazil based on these investigation routines; confirmed cases are classified according to their clinical outcome. During dengue outbreaks, the health system is usually overwhelmed and mild cases may be reported without follow up, but additional information is mandatory for cases with severe outcomes. The results of these investigation efforts by the public health system serve to guide the adoption of control measures and organization of the healthcare network for present and future transmission periods. In our study, the final classification of cases available in SINAN presented a low concordance with the initial diagnosis by physicians at the time of hospitalization. Only 15.1% of those hospitalized as a suspected case of DHF in the public health system met the proposed criteria for this definition; the high proportion (22.1%) of hospitalized DF cases that were reclassified to more severe forms, DwC or ​​DHF, underscore the difficulties of using the WHO protocol in routine epidemiological surveillance [27–29]. Greater accuracy in the identification of severe cases was attempted in SINAN by including classification of Dengue with Complications (DwC). DwC is not a classification option in SIH-SUS. Additionally, the strain of virus, specific sequence of dengue virus infection, comorbidities, the age and possibly the ethnic composition of patient groups may also influence the different patterns observed in different cities and in different years. Better knowledge of the indicators of morbidity and mortality is essential for assessing the burden of dengue and for measuring the impact of intervention strategies [30,31]. The availability of different data sources in Brazil is a most welcoming scenario as it has the potential to increase the representativeness of the surveillance system [32–35]. In this context, the integration of these different sources should be seamless, with automated reports from SIH-SUS to SINAN as suspected cases of mandatorily notifiable diseases are hospitalized. Efforts like this should also be extended to the health insurance companies that also rely on information system for payment purposes.

Other studies have emphasized the need for evaluating the underreporting of hospitalized dengue cases. In Cambodia, data from the routine surveillance system was compared with data from active surveillance; 1.1- to 2.4-fold more hospitalized cases were detected by active surveillance [36]. Similar results were found in another study in Cambodia and Thailand with 1.4 and 2.6-fold more hospitalizations, respectively [37]. In Puerto Rico, the estimated underreporting for inpatients was 42.0% [23]. In Belo Horizonte, Brazil an evaluation of the surveillance system found similar results with a sensitivity of 63% in detecting hospitalized patients [38].

Underreporting of non-hospitalized patients is even more pronounced. The use of active surveillance in Thailand and Cambodia detected 8.7- and 9.1- fold more cases, respectively, than routine surveillance [37]. Other studies showed higher levels of underreporting ranging from 3.9–29 times in Cambodia and 14.0–28 times in Nicaragua [36,39]. The significant undercounting of non-hospitalized patients is likely do to the fact that many infected persons, especially those with non-severe dengue, do not seek healthcare.

The following limitations may have influenced the estimates of sensitivity in our study. We used the data from SIH-SUS as a gold standard, but this system includes only hospitalizations in the public health system, and excludes private healthcare facilities. The private health system is not required to make databases available to research initiatives this was the reason we could not perform an evaluation of underreporting in the private sector. A second potential limitation lies in the methodology of the probabilistic record linkage. Although we have processed a manual review of the pairs with the objective of minimizing errors, it is possible that some pairs were considered true but in fact consisted of different individuals (i.e., false positives), while others may not have been correctly identified (i.e., false negatives) [40].

Although cohort studies are considered the best method for epidemiological estimates of disease incidence, our study confirms the practicality of comparing different databases by using probabilistic methods as a viable alternative for evaluation of surveillance systems. To our knowledge, this is the first study that uses the probabilistic linkage of the databases of SINAN and SIH-SUS in the evaluation of the surveillance of hospitalized dengue cases in multiple cities. Some of our findings reinforce the usefulness of such methodology. The first concerns the revised WHO dengue classification, adopted by Brazil in 2014. Our study may serve as a reference for comparisons in the future on some attributes of the surveillance system employing the new classification. Additionally, the introduction of a dengue vaccine requires a stable, robust surveillance system that provides reliable counts of hospitalized dengue cases, among other indicators, in order to define priority areas and populations for vaccination trials and cost effectiveness studies.

## Supporting Information

S1 Table Number and characteristics of dengue hospitalized cases in the Hospitalization Information System (SIH-SUS) and in the Notifiable Diseases Information System (SINAN) for each of the municipalities of the study, Brazil, 2008–2013. (XLSX)
